# Mixed silages of cactus pear and gliricidia: chemical composition, fermentation characteristics, microbial population and aerobic stability

**DOI:** 10.1038/s41598-020-63905-9

**Published:** 2020-04-22

**Authors:** Gêsica Samíramys Mayra da Silva Brito, Edson Mauro Santos, Gherman Garcia Leal de Araújo, Juliana Silva de Oliveira, Anderson de Moura Zanine, Alexandre Fernandes Perazzo, Fleming Sena Campos, Anny Graycy Vasconcelos de Oliveira Lima, Hactus Souto Cavalcanti

**Affiliations:** 1 Department of Animal Science, Federal University of Paraíba, Areia, Paraíba Brazil; 2 Brazilian Agricultural Research Corporation, EMBRAPA Semiarid, Petrolina, Pernambuco Brazil; 3 Department of Animal Science, Federal University of Maranhão, Chapadinha, Maranhão Brazil; 4 Federal University Rural of Pernambuco, Department of Animal Production, Garanhuns, Pernambuco Brazil

**Keywords:** Biological techniques, Plant sciences

## Abstract

The present study aimed to evaluate the chemical composition, profile and fermentative losses, microbial population and the aerobic stability of mixed silages of cactus pear and gliricidia. The treatments corresponded to the addition levels of gliricidia (*Gliricidia sepium* (Jacq.) Steud), in the silages of cactus pear (*Opuntia ficus indica* Mill.), at ratios 0%, 25%, 50%, 75% and 100% gliricidia. The data were subjected to analysis of variance and regression to evaluate the effect of the addition levels of gliricidia. The average related to the opening days were compared by the Tukey’s test and the average hours of exposure to air were compared by the Student’s t-test. The addition of gliricidia in cactus pear silage provided a linear increasing effect for pH, crude protein (CP), neutral detergent fibre (NDF), acid detergent fibre (ADF), acid detergent lignin (ADL), neutral detergent insoluble protein (NDIP), and increased aerobic stability (AS). The highest dry matter recovery was estimated in the silages with 58% gliricidia. Based on the fermentative, chemical composition and silage losses, all the silages tested were adequate. However, considering aerobic stability, the addition of at least 25% gliricidia is recommended to provide the animal a feed with important quality and high nutritional value.

## Introduction

Both from the productive point of view of the palm and the conservation of the forage nutritional value, cactus pear ensilage would maximize the use of this forage resource, allowing farmers to create a new alternative for the conservation of feed rich in water and energy^1,2^. Cactus pear silage is even more valued for use in feeding ruminants in arid and semi-arid regions, that according to Souza *et al*.^3^ and Borges *et al*.^4^ the inclusion of cactus silage in the ruminant diet reduce water intake, and decrease human-animal competition for water in arid and semi-arid environments where water resources are limited. Furthermore, cactus pear silage allows harvesting of the entire palm planting, standardizing and increasing the regrowth capacity and hence productivity, besides reducing labour with harvest and periodic supply throughout the dry season.

Despite some attributes unfavourable to silage, other characteristics of cactus pear as its bioactive compounds should be considered. The cladodes of the cactus pear are chemically modified structures, composed by chlorophyll and a large percentage of water internally, which exerts the photosynthetic functions of the leaves^5^, as well as the percentage of organic acids found in cladodes, which are oxalic, malic, citric, malonic, succinic and tartaric acid, the latter two being smaller^6^ and showing great variation, especially when evaluated in relation to the planting site and cultural practices.

Another aspect to be evaluated in the cactus pear ensiling process is related to its percentage of water soluble carbohydrates (WSC), since cactus pear is a forage rich in pectic polysaccharides^7^, i.e., esterified sugars with a high concentration of galactose, arabinose, xylose and fructose^8^ that make fermentation possible in the ensilage although showing low contents of dry matter (10 to 13%) and crude protein (4.20 to 6.20%), which prevents it from being recommended as an exclusive food in animal feed^9^.

Another interesting characteristic of the cactus pear is the reduced effluent losses during ensiling due to the formation of a gel in the grinding process of the material that releases cellular constituents of the plant and biochemical transformations that form its gelatinous substance. Such mucilage consists of hydrocolloids distributed throughout the plant and are capable of absorbing water^10,11^ due to its hydrophilic function that minimizes water movement and increases the viscosity of the material, retaining the moisture.

Although it is possible to preserve the palm in the form of silage, as demonstrated in some previous works^5,12,13^, the high amount of sugars and the reduced dry matter content could result in excessive fermentation, which could result in nutrient losses and reduced aerobic stability, since excess sugars may allow proliferation of yeasts^14^, resulting in alcoholic fermentation and, later, reduction of the aerobic stability of the silages.

One of the ways to overcome this problem would be the mixed ensiling of cactus pear with legumes forage, which would result in the buffering of the ensiled mass, reducing the alcoholic fermentation through the inhibition of yeasts. It is known that the main problem associated with legume silage is the high protein content and low soluble carbohydrate content^15^. Among the legumes, gliricidia is highlighted, emerging as an alternative feed for the herds of the semiarid region, with high production of dry matter, reaching 10.7 t/ha in the rainy season and 9.7 t/ha in the dry season, besides desirable nutritional characteristics, such as 30% dry matter and from 20 to 30% crude protein^16–18^. However, in the particular case of the gliricidia, some authors have demonstrated that the final pH values of their silages are always high^19,20^.

The combination of cactus pear with gliricidia would be an alternative to achieve the desired final pH, by predominating the lactic fermentation to the detriment of the alcoholic fermentation, besides improving the silage nutritional characteristics, since the cactus pear shows high content of water-soluble carbohydrates, which are extremely important in the fermentation process. Additionally, the gliricidia shows dry matter and protein with desirable concentrations for the ensiling process. Thus, mixed silage of cactus pear and gliricidia is believed to result in silages with reduced losses, high aerobic stability and, above all, high nutritive value.

In light of the foregoing, the aim of the present study was to evaluate cactus pear silage with the addition of gliricidia on the microbial population, fermentation profile, silage losses, aerobic stability and the chemical composition of the mixed silages to provide the animal a feed with high nutritional value.

## Results

### Fermentation characteristics

The buffer capacity presented by the cactus pear was 14.23 E.g/100 g DM and by the gliricidia of 22.78 E.g/100 g DM (Table 1). The variables pH (P < 0.01), BC (P < 0.01), N-NH_3_ (P < 0.01) and WSC (P < 0.01) had a signification effect with the gliricidia inclusion levels, opening days and interaction between opening days and inclusion levels (Table 2).

**Table 1:** Chemical composition of cactus pear and gliricidia used to produce mixed silages.

Item	Cactus pear	Gliricidia
Dry matter (%)	16.68	24.75
Ash (%DM)	10.16	8.20
Buffer capacity	14.23	22.78
Crude protein (%DM)	4.34	17.92
Total carbohydrates (%DM)	85.46	72.99
Water-soluble carbohydrates	15.06	8.59
Non-fibrous carbohydrates cp (%DM)	59.02	26.04
Ether extract (%DM)	0.04	0.89
N-NH_3_ (%NM)^a^	2.72	5.86
N-NH_3_ (%TN)	4.70	1.75
Neutral detergent fibre (%DM)	26.44	46.59
Acid detergent fibre (%DM)	14.61	33.39
Hemicellulose (%DM)	11.83	13.20
pH	4.95	6.32

^a^% N-NH_3_ on natural matter of the sample; DM, dry matter; NM, natural matter; TN, total nitrogen.

**Table 2:** Effects of gliricidia levels, opening times and the interaction between the factors on fermentation characteristics.

	*p-* value*			
	pH	BC	N-NH_3_	WSC
Levels (L)	<0.001	<0.001	<0.001	<0.001
Opening (O)	<0.001	<0.001	<0.001	<0.001
L × O	<0.001	<0.001	<0.001	<0.001

BC = Buffer capacity; N-NH3 = Ammoniacal nitrogen; WSC = Water-soluble carbohydrates.

*Significant at <0.05.

According to Table 3, the addition levels (25, 50, 75, and 100%) of gliricidia resulted in increments of 57.4, 65.8, 96.4, and 86.3%, respectively, in relation to the average of the buffer capacity of cactus pear silages (15.9 E.g/100 g DM), which explains the increased pH of silages.

**Table 3:** Deployment of the interaction between gliricidia levels and opening times for fermentation characteristics.

Item	Opening	Gliricidia levels					SEM
		0	25	50	75	100	
pH	1	4.29 ± 0.02^a^	4.46 ± 0.08^a^	4.50 ± 0.06^a^	4.69 ± 0.06^a^	6.17 ± 0.07^a^	0.186
	7	3.70 ± 0.02^c^	3.86 ± 0.01^b^	3.96 ± 0.02^b^	4.09 ± 0.04^bc^	5.52 ± 0.19^c^	0.076
	15	3.90 ± 0.09^b^	3.96 ± 0.02^b^	4.09 ± 0.02^b^	4.28 ± 0.05^b^	5.85 ± 0.21^b^	0.197
	30	3.80 ± 0.02^bc^	3.93 ± 0.08^b^	3.98 ± 0.04^b^	4.23 ± 0.08^ab^	5.38 ± 0.11^d^	0.155
	60	3.78 ± 0.05^bc^	3.93 ± 0.05^b^	4.06 ± 0.02^b^	4.18 ± 0.03^abc^	5.16 ± 0.16^e^	0.132
	90	3.68 ± 0.04^c^	3.92 ± 0.06^b^	3.94 ± 0.04^b^	4.02 ± 0.03^c^	4.96 ± 0.07^e^	0.119
Buffer capacity (BC)	1	13.75 ± 0.85^b^	14.73 ± 0.79^d^	17.75 ± 1.74^d^	21.71 ± 0.20^c^	23.74 ± 2.31^c^	0.802
	7	18.55 ± 1.35^a^	20.75 ± 0.85^c^	24.89 ± 2.00^c^	32.00 ± 1.73^b^	34.52 ± 1.34^a^	1.270
	15	14.20 ± 0.52^ab^	26.44 ± 1.39^b^	28.45 ± 0.35^ab^	30.80 ± 1.24^b^	32.49 ± 2.09^b^	1.624
	30	16.34 ± 0.22^ab^	27.94 ± 0.49^ab^	25.85 ± 2.66 ^bc^	33.73 ± 1.13^ab^	33.41 ± 0.90^ab^	1.751
	60	16.42 ± 0.76^ab^	28.64 ± 1.26^ab^	30.11 ± 0.10^ab^	32.45 ± 1.39^ab^	34.16 ± 2.92^a^	2.429
	90	16.16 ± 0.02^ab^	31.69 ± 1.15^a^	31.10 ± 0.08^a^	36.71 ± 0.18^a^	35.41 ± 0.86^a^	1.968
Ammoniacal nitrogen (N-NH_3_)	1	0.84 ± 0.10^c^	3.77 ± 0.48^a^	0.83 ± 0.13^c^	2.50 ± 0.15^c^	3.31 ± 0.16^ab^	0.612
	7	5.89 ± 0.68^a^	3.80 ± 0.60^a^	2.76 ±± 0.18^b^	2.84 ± 0.37^bc^	4.06 ± 0.43^a^	0.566
	15	1.40 ± 0.41^c^	2.37 ± 0.11^b^	2.51 ± 0.07^b^	2.50 ± 0.06^c^	1.82 ± 0.09^c^	0.220
	30	0.43 ± 0.07^c^	2.00 ± 0.10^b^	3.05 ± 0.17^b^	2.87 ± 0.07^bc^	2.97 ± 0.10^abc^	0.496
	60	2.96 ± 0.11^b^	2.47 ± 0.43^ab^	3.09 ± 0.38^b^	4.10 ± 0.37^ab^	2.98 ± 0.40^ab^	0.267
	90	1.01 ± 0.02^c^	3.26 ± 0.45^ab^	8.16 ±± 0.85^a^	4.87 ± 0.48^a^	2.30 ± 0.27^bc^	1.233
Water-soluble carbohydrates (WSC)	1	15.99 ± 0.75^b^	13.67 ± 0.99^a^	12.21 ± 1.23^a^	10.48 ± 0.71^a^	8.46 ± 0.59^a^	0.157
	7	19.84 ± 1.79^a^	11.59 ± 1.07^b^	11.55 ± 0.85^b^	6.47 ± 0.39^ab^	8.04 ± 0.54^a^	2.067
	15	11.64 ± 0.89^b^	10.46 ± 0.69^bc^	11.61 ± 0.89^b^	3.85 ± 0.25^b^	4.05 ± 0.29^b^	1.608
	30	8.75 ± 1.09^bc^	7.44 ± 0.39 ^cd^	10.77 ± 0.79^b^	8.05 ± 0.55^a^	5.58 ± 0.37^ab^	0.757
	90	6.11 ± 0.59^c^	5.53 ± 0.14^d^	5.53 ± 0.49^c^	4.84 ± 0.34^ab^	3.96 ± 0.19^b^	0.329

SEM = standard error of the mean; %TN, Total nitrogen. Averages followed by different letters on the column do not differ among themselves by Tukey’s test (p < 0.05).

### Development of lactic acid bacteria, molds, yeasts and enterobacteria

**Figure 1:**
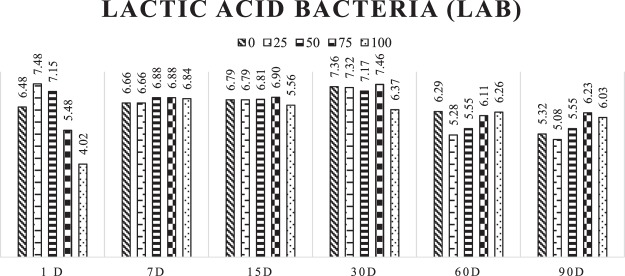
Development of lactic acid bacteria (LAB) in log values at the different opening times of cactus pear silages with addition levels of gliricidia.

The LAB counts with 1 day of silage varied according to the gliricidia levels, but similarities were observed among silages in the opening day 7, with an average of 6.78 (Fig. 1).

At the opening with 90 days, a higher LAB count was observed for treatment with 75% addition of gliricidia, followed by treatments with 100%, 50%, 0%, and 25% addition of gliricidia in the cactus pear silage.

**Figure 2:**
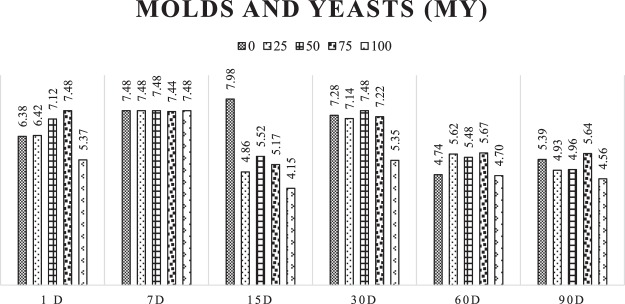
Development of molds and yeasts (MY) in log values at the different opening times of cactus pear silages with addition levels of gliricidia.

The mold and yeast (MY) count with 7 days of opening after ensiling is similar, averaging 7.47. With 90 days of ensiling, the MY number is reduced to 5.39, 4.93, 4.96, 5.64, and 4.56 for the treatments with 0, 25, 50, 75, and 100% addition of gliricidia in cactus pear silage (Fig. 2).

**Figure 3:**
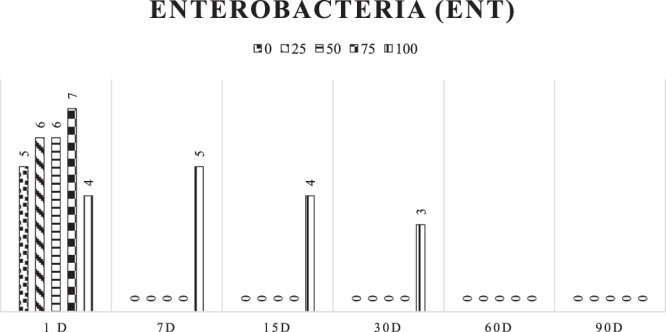
Development of enterobacteria (ENT) in log values at the different opening times of cactus pear silages with addition levels of gliricidia.

The presence of ENT in treatments with 0, 25, 50, and 75% gliricidia added to cactus pear silage with 7 days of opening after ensiling was not observed. For the treatment with 100% gliricidia, the presence of ENT was not observed only in the opening with 60 days (Fig. 3).

**Figure 4:**
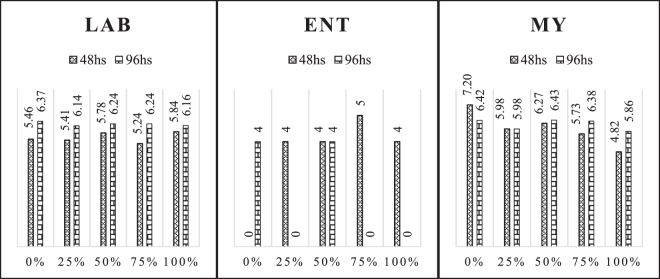
Growth of microorganisms in log values, lactic acid bacteria (LAB), enterobacteria (ENT), molds and yeasts (MY), of cactus pear silages with different addition levels of gliricidia, at times of exposure to air.

The microbial counts LAB, ENT, MY of the cactus pear silages with different addition levels of gliricidia in the times of air exposure are represented by Fig. 4. The LAB presented a higher count with 96 h of exposure to the air for cactus pear silage without addition of gliricidia. In the figure is shown a reduction in the counts of these bacteria as the level of gliricidia in cactus pear silage increases.

The ENT presented similar counts with 48 and 96 h of exposure for treatment with 50% addition of gliricidia in cactus pear silage, but their higher counts were observed with 48 h of air exposure for treatment with addition of 75% gliricidia.

The MY with 48 h of exposure presented higher counts for the treatment without addition of gliricidia and reduced as they added gliricidia in cactus pear silage. At 96 h exposure to air, the treatment with 50% addition of gliricidia, showed a higher count, being reduced with the increase of gliricidia concentration in the silage.

### Organic acid concentrations

Lactic acid (LA), acetic acid (AA), the ratio of lactic acid and acetic acid (LA:AA), and propionic acid (PA) had a signification effect (P < 0.05) with the gliricidia inclusion levels, opening days e interaction. The butyric acid BA had alone an effect with gliricidia inclusion in the cactus pear silage (Table 4).

**Table 4:** Effects of gliricidia levels, opening times and the interaction between the factors for organic acids from cactus pear silages.

	*p*-value*				
	LA	AA	LA:AA	PA	BA
Levels (L)	<0.001	<0.001	<0.001	<0.001	<0.001
Opening (O)	<0.001	<0.001	<0.001	<0.001	0.055
L × O	<0.001	0.002	<0.001	<0.001	0.111

LA = lactic acid; AA = acetic acid; LA:AA = ratio of lactic and acetic acids; PA = propionic acid; BA = butyric acid.

*Significant at <0.05.

The LA concentration showed a negative quadratic effect for almost all opening days, except for the opening with 60 days, which showed a decreased linear as gliricidia was added in the cactus pear silage (Table 5).

**Table 5:** Deployment of the interaction between gliricidia levels and opening times for organic acids content.

Item (% DM)	Opening	Gliricidia levels					SEM
		0	25	50	75	100	
Lactic acid (LA)	1	2.05 ± 0.39^d^	2.87 ± 0.38^b^	3.16 ± 0.53^c^	2.71 ± 0.37^b^	1.36 ± 0.13^b^	0.192
	7	4.94 ± 0.97^c^	4.51 ± 0.76^b^	5.58 ± 1.17^b^	7.05 ± 0.30^a^	5.43 ± 1.16^a^	0.307
	30	6.91 ± 1.30^b^	8.95 ± 0.75^a^	6.15 ± 0.28^b^	6.53 ± 0.07^a^	1.84 ± 0.26^b^	0.685
	60	9.03 ± 0.82^a^	7.22 ± 0.72^a^	6.31 ± 0.29^b^	5.26 ± 0.36^a^	1.67 ± 0.21^b^	0.665
	90	6.05 ± 0.58^bc^	8.27 ± 0.83^a^	8.82 ± 0.30^a^	6.06 ± 0.27^a^	3.06 ± 0.39^b^	0.559
Acetic acid (AA)	1	0.66 ± 0.04^c^	0.81 ± 0.17^b^	0.65 ± 0.10^c^	0.75 ± 0.02^c^	0.28 ± 0.02^b^	0.055
	7	0.68 ± 0.05^c^	0.80 ± 0.07^a b^	1.03 ± 0.18^bc^	1.01 ± 0.19^c^	0.69 ± 0.09^b^	0.056
	30	1.64 ± 0.07^ab^	2.42 ± 0.03^a^	1.85 ± 0.23^a^	1.36 ± 0.03^bc^	1.61 ± 0.14^a^	0.159
	60	1.78 ± 0.18^a^	1.97 ± 0.16^a^	1.31 ± 0.10^abc^	1.24 ± 0.06^bc^	1.42 ± 0.05^a^	0.087
	90	0.97 ± 0.07^bc^	2.20 ± 0.17^a^	1.69 ± 0.09^ab^	1.75 ± 0.08^a^	2.05 ± 0.06^a^	0.122
Ratio of lactic acid and acetic acid (LA:AA)	1	3.11 ± 0.04^b^	3.54 ± 0.20^a^	4.86 ± 0.16^a^	3.61 ± 0.30^b^	4.86 ± 0.02^b^	0.221
	7	7.26 ± 0.06^a^	5.64 ± 0.17^a^	5.42 ± 0.05^a^	6.98 ± 0.17^a^	7.87 ± 0.36^a^	0.496
	30	4.22 ± 0.20^ab^	3.70 ± 0.20^a^	3.32 ± 0.02^a^	4.80 ± 0.13^ab^	1.14 ± 0.05^c^	0.475
	60	5.07 ± 0.15^ab^	3.66 ± 0.16^a^	4.82 ± 0.23^a^	4.24 ± 0.29^b^	1.18 ± 0.03^c^	0.399
	90	6.24 ± 0.14^a^	3.76 ± 0.09^a^	5.22 ± 0.12^a^	3.46 ± 0.05^b^	1.49 ± 0.07^c^	0.432
Propionic acid (PA)	1	0.01 ± 0.00^b^	0.05 ± 0.00^c^	0.04 ± 0.01^b^	0.06 ± 0.01^c^	0.06 ± 0.01^c^	0.005
	7	0.04 ± 0.00^b^	0.06 ± 0.00^c^	0.07 ± 0.01^b^	0.08 ± 0.02^c^	0.08 ± 0.00^c^	0.011
	30	0.08 ± 0.01^ab^	0.26 ± 0.05^ab^	0.24 ± 0.09^a^	0.18 ± 0.05^b^	0.25 ± 0.05^b^	0.021
	60	0.11 ± 0.01^a^	0.22 ± 0.05^b^	0.23 ± 0.05^a^	0.21 ± 0.04^b^	0.24 ± 0.05^b^	0.014
	90	0.09 ± 0.01^ab^	0.31 ± 0.04^a^	0.27 ± 0.09^a^	0.29 ± 0.09^a^	0.38 ± 0.09^a^	0.027

SEM = standard error of the mean. Averages followed by different letters on the column do not differ among themselves by Tukey’s test (p < 0.05).

Regarding the opening days (P < 0.05), the highest LA concentration with 7 days of opening was observed in the treatment with 100% addition and in the treatment with 75% addition of gliricidia, remaining constant up to 90 days of opening. The treatment with 50% gliricidia reached its highest concentration of LA with 90 days of opening, while the treatment with 25% reached it with 30 days of opening and remained constant until the 90 days of opening. In contrast, the treatment without addition of gliricidia only presented a higher concentration of LA at 60 days.

The highest concentration of AA (P < 0.05) with 30 days of opening was found for the treatments with 25, 50 and 100% addition of gliricidia in the cactus pear silage, remaining constant up to 90 days of opening. The treatment without addition of gliricidia showed its highest concentration with 60 days of opening, but statistically similar to the concentration when opened with 30 days; the treatment with 75% gliricidia had its highest concentration of AA at 90 days of opening.

The ratio LA:AA (P < 0.05) showed higher averages with 7 days of opening for all treatments. For treatments with 25 and 50% gliricidia in cactus pear silage, there was no significant difference among the days of opening.

The PA concentration showed higher average values (P < 0.05) for all treatments with addition of gliricidia in the cactus pear silage with opening at 90 days. The treatment without addition of cactus pear had a higher PA concentration with 60 days of opening. The average BA contents reduced linearly (P < 0.01) as gliricidia was added to cactus pear silage (Table 6).

**Table 6:** Butyric acid (BA) content of cactus pear silages with different addition levels of gliricidia.

Item	Gliricidia levels					Equation	*R²*	SEM
	0	25	50	75	100			
BA	0.0145	0.0128	0.0106	0.0092	0.0084	0176 = −0.00006x + 0.01426	67.88	0.002

SEM = standard error of the mean; *R*^2^ = coefficient of determination.

### Losses and fermentation profile

The gas losses (GL, P < 0.01) presented a positive quadratic effect, whereas effluent losses (EL, P = 0.002) showed a decreased linearly as gliricidia inclusion in the cactus pear silage. The dry matter recovery (DMR) was not significantly influenced (P > 0.05) by the addition of gliricidia in the cactus pear silage (Table 7).

**Table 7:** Gas losses (GL), effluent losses (EL) and dry matter recovery (DMR) of cactus pear silages with different levels of gliricidia addition 90 days after ensiling.

Variables	Gliricidia levels					Equation	*R²*	SEM
	0	25	50	75	100			
GL %DM	0.80	0.55	0.46	0.47	0.96	Ŷ = 0.8 − 0.02x + 0.0002x^2^	93.17	0.029
EL kg/ton	23.60	15.51	8.79	2.60	3.74	Ŷ = 5.5221 − 0.055x	98.56	1.853
DMR %	96.60	97.55	98.35	98.85	97.53	Ŷ = 97.84	—	0.217

SEM = standard error of the mean; *R*^2^ = coefficient of determination.

### Chemical composition of silage

The contents of CP, NFC, TC, NDF, ADF, HEM, ADL, NDIP had a signification effect (P < 0.05) with the gliricidia inclusion levels, opening days e interaction between factors. While the DM, OM, EE content had the effect (P < 0.05) alone to gliricidia inclusion levels (Table 8).

**Table 8:** Effects of gliricidia levels, opening times and interaction for the chemical composition mixed silages of cactus pear and gliricidia.

	*p*-value*					
	DM	Ash	OM	EE	CP	NFC
Levels (L)	<0.001	<0.001	<0.001	<0.001	<0.001	<0.001
Opening (O)	0.344	0.075	0.075	0.108	<0.001	<0.001
L × O	0.759	0.378	0.378	0.949	0.016	0.015
	**TC**	**NDF**	**ADF**	**HEM**	**ADL**	**NDIP**
Levels (L)	<0.001	<0.001	<0.001	<0.001	<0.001	<0.001
Opening (O)	<0.001	<0.001	<0.001	<0.001	<0.001	<0.001
L × O	<0.001	<0.001	<0.001	<0.001	<0.001	<0.001

DM = dry matter, OM = organic matter, EE = ether extract, CP = crude protein, NFC = non-fibrous carbohydrates, TC = total carbohydrates, NDF = neutral detergent insoluble fibre, ADF = insoluble acid detergent fibre, HEM = hemicellulose, ADL = Acid detergent lignin, NDIP = Neutral detergent insoluble protein.

*Significant at <0.05.

Concentrations of dry matter (DM, P < 0.01), organic matter (OM, P < 0.01) and ether extract (EE, P < 0.01) increased linearly, while ash concentration linearly reduced (P < 0.01) with the addition of gliricidia in cactus pear silage (Table 9).

**Table 9:** Dry matter (DM), ash, organic matter (OM) and ether extract (EE) of the mixed silages of cactus pear and gliricidia.

Item	Gliricidia levels					Equation	*R*^2^	SEM
	0	25	50	75	100			
DM	15.59	18.11	20.75	23.56	24.09	Ŷ = 15.902 + 0.0904x	90.55	0.270
Ash	11.34	10.07	9.80	9.20	8.75	Ŷ = 11.044 − 0.0242x	81.02	0.061
OM	88.66	89.93	90.20	90.80	91.25	Ŷ = 88.956 + 0.0242x	81.02	0.062
EE	0.95	1.37	1.62	2.06	2.73	Ŷ = 0.8973 + 0.0169x	43.56	0.095

SEM = standard error of the mean; *R*^2^ = coefficient of determination.

Content of CP, NDF, HEM, and NDIP increased (P < 0.05) with gliricidia inclusion in silage and had higher averages with 15 opening days. While ADF and ADL contents had the same behaviour at 30 and 90 opening days, respectively. NFC and TC contents decreased (P < 0.05) with gliricidia inclusion in silage and had higher averages with 15 and 7 opening days, respectively (Table 10).

**Table 10:** Deployment of the interaction between gliricidia levels and opening time for chemical composition of mixed cactus pear silages and gliricidia.

Item	Opening	Gliricidia levels					SEM
(%DM)		0	25	50	75	100	
Crude protein (CP)	1	4.29 ± 0.36^a^	7.85 ± 0.36^c^	11.57 ± 0.01^a^	13.34 ± 1.07^a^	15.48 ± 0.35^b^	1.335
	7	3.95 ± 0.38^a^	9.62 ± 1.10^abc^	12.56 ± 0.36^a^	14.37 ± 0.72^a^	18.05 ± 0.01^ab^	1.589
	15	6.55 ± 0.86^a^	9.95 ± 0.01^abc^	11.75 ± 1.83^a^	14.31 ± 0.36^a^	19.07 ± 1.01^a^	1.436
	30	8.53 ± 1.27^a^	12.41 ± 0.86^a^	13.77 ± 0.01^a^	15.51 ± 0.37^a^	16.32 ± 0.74^ab^	0.951
	60	6.38 ± 0.02^a^	9.41 ± 0.71^bc^	11.23 ± 0.36^a^	13.51 ± 0.02^a^	17.42 ± 0.34^ab^	1.249
	90	7.10 ± 1.11^a^	11.77 ± 1.10^ab^	12.60 ± 0.76^a^	13.58 ± 2.01^a^	18.10 ± 1.09^ab^	1.217
Non-fibrous carbohydrate (NFC)	1	60.66 ± 0.87^a^	50.69 ± 1.29^a^	39.92 ± 1.15^a^	35.76 ± 1.75^ab^	30.39 ± 1.33^a^	3.636
	7	60.19 ± 2.45^a^	50.01 ± 1.74^a^	41.67 ± 2.78^a^	38.23 ± 1.11^a^	28.15 ± 0.81^a^	3.656
	15	62.04 ± 2.03^a^	48.29 ± 1.09^ab^	41.77 ± 2.25^a^	36.02 ± 1.79^ab^	24.32 ± 0.29^a^	4.214
	30	59.77 ± 2.54^a^	46.05 ± 0.69^abc^	42.68 ± 0.47^a^	36.87 ± 0.21^ab^	28.93 ± 0.61^a^	3.431
	60	55.04 ± 0.66^a^	44.66 ± 1.57^bc^	40.73 ± 0.78^a^	31.90 ± 1.05^b^	27.76 ± 0.37^a^	3.219
	90	56.84 ± 2.16^a^	42.39 ± 0.72^bc^	38.54 ± 0.15^a^	33.72 ± 2.34^ab^	25.81 ± 1.08^a^	3.454
Total carbohydrates (TC)	1	82.88 ± 0.68^ab^	80.11 ± 0.36^a^	71.31 ± 0.68^a^	75.00 ± 1.30^a^	72.73 ± 0.02^a^	1.212
	7	84.15 ± 0.09^a^	79.15 ± 0.77^abc^	76.92 ± 0.21^a^	74.88 ± 0.69^a^	70.52 ± 0.31^ab^	1.511
	15	81.70 ± 2.25^ab^	79.56 ± 0.44^ab^	76.63 ± 2.05^a^	74.94 ± 0.20^a^	68.58 ± 2.19^b^	1.553
	30	79.78 ± 2.39^b^	75.97 ± 0.85^c^	74.58 ± 0.06^a^	73.35 ± 0.20^a^	73.18 ± 0.03^a^	0.850
	60	80.87 ± 0.06^ab^	79.03 ± 0.81^abc^	76.98 ± 0.47^a^	73.96 ± 1.35^a^	70.48 ± 0.65^ab^	1.244
	90	80.04 ± 1.10^b^	76.57 ± 0.72^bc^	75.61 ± 0.65^a^	75.73 ± 2.32^a^	71.22 ± 1.03^ab^	0.988
Neutral detergent insoluble fibre (NDF)	1	22.72 ± 0.31^bc^	30,23 ± 0.97^b^	38.75 ± 1.77^a^	42.46 ± 0.36^ab^	46.72 ± 1.19^a^	2.893
	7	24.61 ± 2.54^ab^	30.75 ± 0.94^b^	37.24 ± 2.69^ab^	39.67 ± 0.43^b^	46.25 ± 3.25^a^	2.536
	15	20.37 ± 0.16^c^	33.06 ± 0.57^b^	37.56 ± 0.18^ab^	42.29 ± 1.60^ab^	49.04 ± 0.50^a^	3.216
	30	20.95 ± 0.21^bc^	32.18 ± 0.01^ab^	34.95 ± 0.57^b^	40.43 ± 0.02^b^	48.75 ± 0.79^a^	3.068
	60	26.51 ± 0.61^a^	36.74 ± 0.62^a^	39.29 ± 1.29^a^	45.65 ± 0.36^a^	46.49 ± 0.27^a^	2.417
	90	23.60 ± 1.05^abc^	36.27 ± 0.01^a^	39.48 ± 0.77^a^	45.17 ± 0.20^a^	49.18 ± 2.22^a^	2.942
Acid detergent insoluble fibre (ADF)	1	12.29 ± 0.06^bc^	19.61 ± 0.39^c^	26.46 ± 1.14^c^	28.96 ± 0.96 ^cd^	34.48 ± 0.15^c^	2.572
	7	12.48 ± 1.10^bc^	18.59 ± 0.06^c^	26.64 ± 0.97^bc^	28.14 ± 0.67^d^	32.20 ± 0.96^d^	2.377
	15	11.59 ± 0.28^c^	24.15 ± 0.37^b^	26.70 ± 0.14^abc^	30.91 ± 0.48^bc^	33.73 ± 0.68 ^cd^	2.557
	30	12.28 ± 0.24^bc^	23.95 ± 0.32^b^	25.87 ± 0.97^c^	31.16 ± 0.76^b^	37.79 ± 0.80^a^	2.824
	60	15.13 ± 0.67^a^	27.03 ± 0.37^a^	28.58 ± 0.85^ab^	33.67 ± 0.57^a^	35.44 ± 0.88^bc^	2.383
	90	13.78 ± 0.19^ab^	26.62 ± 0.01^a^	28.78 ± 0.25^a^	34.07 ± 0.15^a^	37.21 ± 1.62^ab^	2.698
Hemicellulose (HEM)	1	10.43 ± 0.24^ab^	10.62 ± 0.57^ab^	12.29 ± 0.64^a^	13.50 ± 0.60^a^	12.24 ± 1.35^bc^	0.423
	7	12.13 ± 1.44^a^	12.16 ± 0.96^a^	10.60 ± 1.72^ab^	11.53 ± 0.25^abc^	14.05 ± 2.29^ab^	0.517
	15	8.78 ± 0.12^b^	8.91 ± 0.20^b^	10.86 ± 0.06^ab^	11.38 ± 1.11^abc^	15.31 ± 0.18^a^	0.798
	30	8.67 ± 0.03^b^	8.23 ± 0.31^b^	9.08 ± 0.39^b^	9.27 ± 0.75^c^	10.96 ± 0.06^c^	0.324
	60	11.38 ± 0.16^a^	9.71 ± 0.26^b^	10.71 ± 0.45^ab^	11.98 ± 0.21^ab^	11.05 ± 0.61^c^	0.266
	90	9.82 ± 0.86^ab^	9.65 ± 0.07^b^	10.70 ± 0.52^ab^	11.10 ± 0.35^bc^	11.97 ± 0.60^bc^	0.313
Acid detergent lignin (ADL)	1	0.015 ± 0.001^ab^	0.026 ± 0.001^b^	0.037 ± 0.004^bc^	0.046 ± 0.001^ab^	0.064 ± 0.003^a^	0.006
	7	0.012 ± 0.001^b^	0.023 ± 0.001^b^	0.036 ± 0.004^c^	0.043 ± 0.001^c^	0.056 ± 0.001^b^	0.005
	15	0.013 ± 0.001^b^	0.035 ± 0.001^a^	0.043 ± 0.002^ab^	0.048 ± 0.001^ab^	0.056 ± 0.001^b^	0.005
	30	0.013 ± 0.001^b^	0.037 ± 0.003^a^	0.043 ± 0.001^ab^	0.051 ± 0.002^b^	0.063 ± 0.001^a^	0.006
	60	0.020 ± 0.001^a^	0.041 ± 0.001^a^	0.047 ± 0.005^a^	0.058 ± 0.002^a^	0.064 ± 0.001^a^	0.005
	90	0.016 ± 0.001^ab^	0.042 ± 0.002^a^	0.049 ± 0.001^a^	0.059 ± 0.002^a^	0.067 ± 0.004^a^	0.006
Neutral detergent insoluble protein (NDIP)	1	0.49 ± 0.02^bc^	0.81 ± 0.03^d^	1.35 ± 0.05^e^	3.23 ± 0.10 ^cd^	4.38 ± 0.16^b^	0.501
	7	0.66 ± 0.01^bc^	1.61 ± 0.04^c^	1.99 ± 0.13^d^	3.03 ± 0.02^d^	3.87 ± 0.06^c^	0.373
	15	0.71 ± 0.07^ab^	1.79 ± 0.08^c^	2.70 ± 0.02^b^	3.38 ± 0.10^bc^	4.78 ± 0.05^a^	0.462
	30	0.94 ± 0.02^a^	2.26 ± 0.15^ab^	3.05 ± 0.08^a^	3.95 ± 0.12^a^	4.51 ± 0.15^b^	0.421
	60	0.68 ± 0.07^ab^	2.38 ± 0.09^a^	3.04 ± 0.11^a^	3.58 ± 0.07^b^	3.77 ± 0.07^c^	0.372
	90	0.40 ± 0.01^c^	2.10 ± 0.02^b^	2.42 ± 0.01^c^	3.15 ± 0.02 ^cd^	3.77 ± 0.18^c^	0.382

SEM = standard error of the mean. Averages followed by lowercase letters on the same column do not differ among themselves by Tukey’s test (p < 0.05).

There was no significant difference (P > 0.05) between the opening days for the CP and NFC nutritional fractions at the 0 and 50% levels of gliricidia.

### Silage aerobic stability

The pH had a signification effect (P < 0.05) with the gliricidia inclusion levels, time of exposure to air e interaction between factors. While the BC had the effect (P < 0.05) alone to gliricidia inclusion levels (Table 11).

**Table 11:** Effects of gliricidia levels, time of exposure to air (TA) and interaction between the factors for pH and buffer capacity (BC) in cactus pear silages with addition levels of gliricidia.

	p-value*	
	pH	BC
Levels (L)	<0.001	<0.001
Time of exposure to air (TA)	0.001	0.109
L × TA	0.003	0.057

*Significant at <0.05.

The pH presented a quadratic positive effect (P < 0.01) with the addition of gliricidia in cactus pear silage for 48 and 96 hours of exposure to air (Table 12). When the exposure time was evaluated, treatments that included gliricidia had higher mean values with 48 h of exposure, while treatment without addition had a higher mean with 96 h of exposure (Table 12).

**Table 12:** pH of the cactus pear silages with different levels of gliricidia addition during air exposure times and BC, temperature and aerobic stability (AS).

Variables	Gliricidia levels					Equation	*R²*	SEM
	0	25	50	75	100			
pH								
48 h	4.68 ± 0.25b	3.92 ± 0.06a	3.98 ± 0.04b	4.11 ± 0.04a	5.01 ± 0.04a	Ŷ = 4.7 − 0.04 × 0.0004x²	96.86	0.133
96 h	5.99 ± 0.13a	4.38 ± 0.15a	4.49 ± 0.12a	4.05 ± 0.01a	5.00 ± 0.01a	Ŷ = 5.9 − 0.06x + 0.001x²	88.40	0.183
BC	7.81 ± 0.91	25.69 ± 4.12	29.16 ± 3.91	37.35 ± 4.01	46.54 ± 5.51	Ŷ = 11.189 + 0.3684x	91.03	0.613
Max temp (°C)	32.77 ± 0.84	29.67 ± 2.86	29.63 ± 1.15	27.97 ± 0.78	27.07 ± 0.38	Ŷ = 32.04 − 0.0524x	65.36	0.072
AS (h)	26.67 ± 5.21	57.67 ± 7.57	65.33 ± 11.73	71.67 ± 14.04	83.33 ± 13.51	Ŷ = 36.067 + 0.5013x	63.77	20.07

SEM = standard error of the mean; *R*^2^ = coefficient of determination.

Averages followed by lowercase letters on the same column do not differ among themselves by t-test (*P* < 0.05)

The linear effect was shown to be increasing in the buffer capacity (P < 0.01) and aerobic stability (P < 0.01) and decreasing linear effect for the maximum temperature (P < 0.01) of cactus pear silages with addition of gliricidia. The higher values of the buffer capacity for silages with a higher proportion of gliricidia justify the lower pH change of the silages at times of exposure to air.

## Discussion

The addition of gliricidia in cactus pear silage provided a linear increase in pH for all opening days, which may be justified by the higher buffer capacity, as well as the lower soluble carbohydrate intake of gliricidia, since these characteristics affect the pH drop which will be explained in more detail below.

The pH values of the cactus pear-containing silages coincide with the values found by Nogueira *et al*.^21^, which evaluated the potential of the cactus pear for silage, without and with additives (wheat bran and urea) and obtained values of 3.8 and 4.2. These values are considered ideal for well-fermented silages^22^.

The legumes generally have resistance to pH reduction in silages due to their high buffering capacity, mainly due to their presence of cations (K^+^, Ca^2+^ and Mg^2+^). These cations come into contact with organic acids formed by the fermentation, neutralizing them, and preventing the occurrence of pH drop^23^.

In relation to the water-soluble carbohydrates (WSC), the addition of gliricidia in the cactus pear silages provided generally a reduction in the contents according to the openings.

The main problem associated with legume silage is the low water-soluble carbohydrate content, since the cactus pear has enough water-soluble carbohydrates. However, the low DM content associated with the low CP content creates the demand for the addition of legumes like gliricidia with high CP content and adequate DM content. Gusha *et al*.^12^ state the cactus pear has a high concentration of water-soluble carbohydrates which, in turn, allows a rapid decline the pH to a silage preservation range.

The amount of ammoniacal nitrogen present in the silages is a form of observation of proteolytic activity, being an indirect indicator of clostridial activity, and that may contribute to the elevation of silage pH^24^. According to McDonald *et al*.^22^, high concentrations of total N-NH_3_ are above 10% and in general these high concentrations are due to the slow fall of the pH in general and indicate poorly fermented silages. The concentrations of ammoniacal nitrogen in percentage total nitrogen of the dry matter (N-NH_3_% TN) were below the 10% recommended by McDonald *et al*.^22^, which is indicative that there was no excessive protein breakdown in ammonia, characterizing adequate fermentation of the silages.

Regarding the microbial populations, the lowest number of LAB was observed in the cactus pear before silage, about 5.98 log CFU/g, whereas the one found in the gliricidia before silage was 6.81 log CFU/g.

The cactus pear and the gliricidia showed close values of molds and yeasts, averaging around 5.87 and 5.91 UFC/g of plant, respectively. Despite the rapid production of lactic acid and reduction of pH, occurring due to the amount of water-soluble carbohydrates available, the population of molds and yeasts was present in all silages and even during 90 days of silage, possibly due to their capacity to be developed at low pH.

According to Muck^25^, these microorganisms can grow at pH ranging from 2 to 9. It is worth mentioning that the number of molds and yeasts was slightly higher than the number of lactic acid bacteria up to 30 days of silage, with a decrease of this population from 60 days of silage. Once molds and yeasts are facultative aerobic, this allowed their proliferation in the first days of silage, and the large availability of soluble sugars that, in a large amount, inhibit of the proliferation of gram-positive bacteria, such as LAB and ENT after 60 days of silage. Silage’ conditions favoured the rapid development of LAB and reduced the population of molds and yeasts from 60 days of silage.

In plants, it was possible to detect the presence of enterobacteria, about 6 log CFU/g in both cactus pear and gliricidia. Only on the 1st day of silage, it was possible to verify the presence of enterobacteria in the silages containing cactus pear. In contrast, the gliricidia silages presented enterobacteria up to 30 days after ensiling. This fact is explained by the higher buffer capacity and hence lower reduction of pH for the gliricidia silages.

The ENT are a group of bacteria that ferment the water-soluble carbohydrates to acetic acid. Such acid is common in the fermentation process and is desirable in the fermentative process due to its antifungal action. However, some species of this group of bacteria can also be undesirable for degrading part of the protein, which reflects in the formation of ammonia and reduction of nitrate, which hinder the accentuated drop of the pH^22,26^.

The lactic acid values of the present study were higher than the values found by Çürek and Özen^27^, who found average levels of 2.59% and 3.20% lactic acid when evaluating silage of young and old cladodes of cactus pear, respectively. In the exclusive silage of gliricidia, a lower concentration of lactic acid was observed, 3.06% in the opening of 90 days after ensiling. In the literature is reported a direct relationship between the lactic acid concentration and the pH of the silage, since the pKa of lactic acid is the lowest of the other organic acids produced in the silage process, thus increasing its capacity to reduce the pH when compared to acetic acid.

In relation to the average content of acetic acid (AA) found in the evaluated silages, variation in the average values with the addition of gliricidia is observed. These values are close to the ones found by Oduguwa *et al*.^28^, which was about 1.13 and 1.42% AA in DM; and similar to those found by Çürek and Özen^27^, around 1.53 and 1.52% AA in DM when evaluating cactus pear silages with young cladodes and silages with mature cladodes. The higher values of acetic acid can be explained by the presence of enterobacteria and possibly by the presence of heterofermentative LAB.

The ratio of lactic acid and acetic acid was not affected, except for the opening with 1 day after silage that showed a quadratic effect (Ŷ = 3.1405 + 0.0257x − 0.0001x^2^). gliricidia silage presented a lower ratio of lactic acid and acetic acid (1.57%), indicating lower lactic acid production in the silage and likely more stable when exposed to air. Kung Junior *et al*.^29^ reported that moderate concentrations of acetic acid in silage may be beneficial as they inhibit yeasts, resulting in increased aerobic stability.

The content of propionic acid increased from 30 days of silage, with the concentration of this acid higher in the gliricidia silages at the end of the process (0.38% DM). This concentration is considered ideal according to Roth and Undersander^30^, since they state that propionic acid lower than 0.50% is indicative of well-fermented silages. Conversely, propionic acid has a high antifungal action, which may favour the increase of the aerobic stability of silages.

The addition levels of gliricidia in the silages were influenced in the average contents of butyric acid (BA); however, the variation was so minimal as to be insignificant. This shows that the silage was well fermented once the values are in the range recommended as ideal, which is less than or equal to 1%.

Gas losses showed a quadratic positive effect, with minimum values between the addition levels 50 and 75% gliricidia. Due to the higher DM content of the gliricidia in relation to the cactus pear, the silages showed lower effluent losses inasmuch as gliricidia was added; however, the level with 75% showed the lowest value (2.60 kg/ton). The value for the effluent losses of the cactus pear silage is similar to the found by Nogueira *et al*.^21^, of 23.06 kg/ton of silage. Driehuis and Wikselaar^31^ state that cactus pear mucilage is effective in retaining fluids of the ensiled masses, thus reducing linearly as the concentration of gliricidia in cactus pear silage was increased.

The DMR was not influenced by the gliricidia added. However, with the reduced production of effluent and control of undesired fermentation, the complementary effect of the two forages studied is emphasized, so that mixed silage results in lower losses during the ensiling, corroborating the hypothesis of the present study.

The effect of addition levels of gliricidia on dry matter (DM), ash, organic matter (OM) and ether extract (EE) was verified. However, there was no interaction effect between the factors studied. Gliricidia has a higher content of DM, MO, and EE than cactus pear. In this way, these nutritional fractions increased with gliricidia inclusion in the silages.

There were increasing linear for CP, NDF, ADF, ADL, NDIP, and linear decreasing effect for NFC and TC. Mixed silages are influenced by the nutritional value of each forage. As verified by the interaction, there was a decrease of the non-fibrous carbohydrates as a function of the opening times due to the fermentation of sugars during the fermentation period. Thus, by dilution, this effect influenced other variables of the chemical composition of silages.

When oxygen enters the silo during silage production, or during silage supply to animals, the multiplication of some groups of aerobic or aerobic facultative microorganisms are favoured, especially molds and yeasts, and some proteolytic bacteria that use energy silage^32^, which increases the losses of DM and nutritional value, negatively affecting the productive performance of the animals.

The cactus pear silage at 90 days of silage had a pH of 3.68. However, during exposure to air during 48 and 96 h, the pH increased, ranging from 4.68 to 5.99. This increase may have occurred due to the proliferation of yeasts that use lactic acid, decreasing their amount in the medium and favouring the increase of pH^25^. This occurs in all silages evaluated in this experiment, during exposure to air, when compared to pH values at 90 days of silage, but in a less pronounced level.

The variation in the durability of silages is also linked to the development of microorganisms. It is observed that the LAB number did not vary considerably, averaging 5 to 6 log CFU/g of silage, from 48 to 96 h of silage exposure to air. This indicates a post-opening activity, mainly because it has sufficient substrate.

Growth of enterobacteria was verified when exposed to air, being more pronounced during the first 48 h, from 3.7 decreasing to about 1.6 log CFU/g of silage. This behaviour of decreased population can be justified by the high competition with the other groups present in the ensiled mass, which limited the number of substrates available for these groups.

The development of molds and yeasts was more pronounced in the silages with less addition of gliricidia and during the first 48 h of exposure to air, which can be explained by the lower values of acetic acid. It is evident that the addition of gliricidia provides production of acetic acid, which is fundamental in controlling fungi after exposure to air.

This group, through respiration, causes losses of energy and nutrients by the release of CO_2_ and heat (loss of energy) and effluents (loss of nutrients). It may explain why silages showed the highest temperatures during aerobic stability.

The addition of gliricidia in cactus pear silage provided increased aerobic stability; however, no silage remained stable throughout the evaluated period (96 h). On the other hand, it was observed that all silages with gliricidia presented high aerobic stability, above 70 h, which can be attributed to higher production of acetic acid.

The mixed silages of cactus pear and gliricidia show adequate fermentative, nutritional and exposure to air response characteristics, which are indicative of an efficiently preserved silage in all aspects of the ensiling process. This fact points to a possibility of utilizing and optimizing the use of these two very important forage resources for the arid and semiarid regions, allowing both to be harvested with high nutritional value, bypassing a problem already known related to legume silage and allowing the harvesting and ensilage of cactus pear, optimizing the use and its agronomic potential. Further studies are necessary in order to evaluate the intake, digestibility and performance of animals fed with diets based on mixed silages of cactus pear and gliricidia.

Thus, based on the fermentative profile, chemical composition and silage losses, all the silages tested were adequate. However, considering aerobic stability, the addition of at least 25% gliricidia is recommended to provide the animal a feed with important quality and high nutritional value.

## Methods

### Location and meteorological data

The experiment was performed at the premises of the Brazilian agricultural research agency (EMBRAPA Semi-arid), located in the municipality of Petrolina, PE, Brazil, at the geographic coordinates 09°04′16.4″S and 40°19′5.37″W, 379 m altitude. The average annual rainfall is 570 mm and the maximum and minimum temperatures are 33.5 and 20.9 °C, respectively.

Analyses were partly performed in the animal nutrition laboratory of the same institution and partly in the Forage laboratory of the Forage Sector, belonging to the Animal Science Department of the Federal University of Paraíba (UFPB), Campus II, Areia-PB.

### Silages production

The gliricidia was collected in the experimental field of the Embrapa semi-arid, which was already being managed for regular (semi-annual) cutting. The aerial part of the plant was selected, represented by more tender leaves and stems. The cactus pear was also harvested in an experimental field of the same institution, where the last cut was made approximately two years ago. The cut cladodes were harvested and then minced into a forage machine approximately 2.5 × 2.5 cm in size. After this process, samples of the two forage plants were collected, where part was used to evaluate the natural material and part of the samples was stored for further analysis.

A total of 90 polyvinyl chloride (PVC) silos were used, in which 2 kg of absorbent material (coarse sand) were placed in the bottom of each silo in order to absorb the possible effluents from the fermentation process. Above this absorbent layer, a piece of synthetic fibre of the nonwoven type (NWT) was used, with the function of separating the sand from the silage, thus avoiding contamination of the same. After this, about 4 kg of forage were compacted in the silos, according to the proportion of each treatment. After compaction, the silos were sealed with plastic caps that contained Bunsen valves, thus favouring the exit of gases produced during the fermentation process.

The treatments corresponded to the addition levels of gliricidia (*Gliricidia sepium* (Jacq.) Steud), in the silages of cactus pear (*Opuntia ficus indica* Mill.), at ratios 0%, 25%, 50%, 75% and 100% gliricidia.

The chemical composition of the forage plants used to make the silages can be observed in Table 1.

### Fermentation characteristics and microbial population

For the analyses of the fermentation profile and microbial populations, the central parts of the ensiled mass were used, being discarded approximately 5 cm of each (upper and lower) portion of each silo, well homogenizing the fraction collected.

The analysed variables of the fermentation process were performed at each opening time (1, 7, 15, 30, 60, 90 days). The values of potential of hydrogen (pH), as well as the microbiological analysis, were evaluated by counting the microbial populations, being lactic bacteria (LAB), enterobacteria (ENT), molds and yeasts (MY). The content of organic acids, such as lactic acid (LA), acetic acid (AA), propionic acid (PA) and butyric acid (BA); and ammoniacal nitrogen content in relation to the rate of total nitrogen (N-NH_3_-%TN); buffer capacity (BC) and water soluble carbohydrates (WSC) were also analysed.

To determine pH, a 25 g silage sample was used, following the methodology described by Bolsen *et al*.^33^. The silage was homogenized in 100 mL of distilled water, which remained standing for 1 h for pH reading, using a pH meter.

The microbial populations were quantified in the mixed silages prior to ensiling, and in the silages using selective culture media for each microbial group: MRS (Man, Rogosa and Sharpe) Agar containing 0.4% nystatin for the LAB; Violet Red Bile Agar for the ENT; and Potato Dextrose Agar, containing 1% of 10% tartaric acid for the MY the methodology described by Bezerra *et al*.^34^.

The microbial groups were quantified from 10 g of a sample composed of the replicates of each silage in which 90 mL of sterilized distilled water were added and homogenized for 1 minute, obtaining a dilution of 1/10. Then, successive dilutions were performed aiming to obtain dilutions ranging from 1/10 to 1/1000000000. The culture of the microorganisms was performed in sterile disposable Petri dishes. The plates were incubated according to specific temperatures for each microbial group, LAB, 37 °C for 48 h; ENT, 30 °C for 24 h, and MY, 28 °C for 72 h^35,36^. Plaques with values between 30 and 300 colony forming units (CFU) were considered countable.

Organic acids (lactic, acetic, propionic and butyric) were determined using the methodology described by Kung Junior and Ranjit^37^. The analysis was performed on a High-Performance Liquid Chromatograph (HPLC), brand SHIMADZU, model SPD-10A VP coupled to the Ultraviolet Detector (UV) using a wavelength of 210 nm

The N-NH_3_ and BC of the silages were performed following the methodologies described by Bolsen *et al*.^33^ and Playne and McDonald^38^, respectively.

The amount of water-soluble carbohydrates (WSC) was determined according to Dubois *et al*.^39^ with adaptations of Corsato *et al*.^40^ using concentrated sulfuric acid.

The DM recovery (DMR) was estimated according to Zanine *et al*.^41^. The effluent (EL) gases (GL) and losses were quantified using the following equation proposed by Jobim *et al*.^42^.

### Aerobic stability

The aerobic stability (AS) of the silages (expressed in hours) was evaluated by monitoring the temperatures (superficial and internal) of the silages exposed to air.

The silage samples were placed without compaction in experimental silos of uncovered PVC, kept in a closed environment with controlled temperature (25 °C). Up to 96-h experimental period of aerobic stability evaluation was considered, assessing microbial populations, fermentation profile, chemical composition and DM recovery of silages at 24, 48 and 96 h of exposure to air.

The temperatures were verified every hour, using thermometers (digital laser and digital immersion) positioned in the centre of the silage mass. The beginning of deterioration was considered when the internal temperature of the silages reached 2 °C above room temperature^43^.

### Chemical composition

From the plant samples, the percentages of dry matter (DM, method 967.03), ash (method 942.05), crude protein (CP, method 981.10) and ether extract (EE, method 920.29) were determined according to AOAC^44^.

The neutral detergent fibre content corrected for ash and protein (NDFap)^45,46^ and acid detergent fibre (ADF) were determined as described by Van Soest *et al*.^47^. Acid detergent lignin (ADL) was determined by treating the acid detergent fibre residue with 72% sulfuric acid^48^.

To estimate the total carbohydrates (TC), the Eq. (1) proposed by Sniffen *et al*.^49^ was used:

1 $$TC=100-( \% CP+ \% EE+ \% Ash)$$

The non-fibrous carbohydrates (NFCap) were estimated using the equations recommended by Hall^50^ according to Eq. (2)

2 $$NFCap= \% TC- \% NDFap$$

### Statistical analyses

In order to evaluate the characteristics associated to the fermentation process, the experimental design was completely randomized, with a 5 × 6 factorial design and three replicates, where the five addition levels of gliricidia (0, 25, 50, 75 and 100%) in cactus pear silage and six silo opening times (1, 7, 15, 30, 60 and 90 days) were evaluated. The effect of variables isolated was not evaluated when the interaction was significant.

For the aerobic stability test, the same completely randomized design with a 5 × 2 factorial design and three replicates was used to evaluate the five addition levels of gliricidia (0, 25, 50, 75 and 100%) in cactus pear silage and two evaluation times (48 and 96 h of exposure to air) by t-test at 0.05 probability.

The data were subjected to analysis of variance and polynomial contrasts were used to determine the linear and quadratic effects of the different levels of gliricidia, through the statistical software SAS 9.1 (SAS Institute, Cary, NC, USA). *p*-values less than 0.05 were considered significant.

The most appropriate model for each variable was chosen based on the significance of the linear and quadratic coefficient of determination. The averages for the opening days were compared by Tukey’s test and the average hours of exposure to air were compared by Student’s t test, both at 0.05 probability.
